# Associations between Urinary Excretion of Cadmium and Renal Biomarkers in Nonsmoking Females: A Cross-Sectional Study in Rural Areas of South China

**DOI:** 10.3390/ijerph121011988

**Published:** 2015-09-24

**Authors:** Yun-rui Zhang, Ping Wang, Xu-xia Liang, Chuen Seng Tan, Jian-bin Tan, Jing Wang, Qiong Huang, Rui Huang, Zhi-xue Li, Wen-cai Chen, Shi-xuan Wu, Choon Nam Ong, Xing-fen Yang, Yong-ning Wu

**Affiliations:** 1 Guangdong Provincial Center for Disease Control and Prevention, Guangzhou 511430, China; E-Mails: jnurae@163.com (Y.Z.); wangp@gdiph.org.cn (P.W.); liangxuxia@126.com (X.L.); tan-sword@163.com (J.T.); wjgdcdc@126.com (J.W.); huangqiong@cdcp.org.cn (Q.H.); huangr@gdiph.org.cn (R.H.); jnulizhixue@163.com (Z.L.); chenwcgz@126.com (W.C.); wshixuan@126.com (S.W.); 2 Department of Occupational and Environmental Health, Medical School, Ji’Nan University, Guangzhou 510632, China; 3 Guangdong Provincial Institute of Public Health, Guangzhou 511430, China; 4 Saw Swee Hock School of Public Health, National University of Singapore, 119077, Singapore; E-Mail: ephtcs@nus.edu.sg; 5 National University of Singapore Environmental Research Institute (NERI), 117597 Singapore; E-Mail: choon_nam_ong@nuhs.edu.sg; 6 Key Laboratory of Food Safety Risk Assessment of Ministry of Health, China National Center for Food Safety Risk Assessment, Beijing 100022, China

**Keywords:** cadmium, biomonitoring, renal, women, cross-sectional studies

## Abstract

*Objectives:* The aim of this study was to systematically evaluate the relationship between urinary excretion of cadmium (U-Cd) and biomarkers of renal dysfunction. *Methods:* One hundred eighty five non-smoking female farmers (aged from 44 to 71 years) were recruited from two rural areas with different cadmium levels of exposure in southern China. Morning spot urine samples were collected for detecting U-Cd, urinary creatinine (U-*cre*), β_2_-microglobulin (β_2_-MG), α_1_-microglobulin (α_1_-MG), metallothionein (MT), retinol binding protein (RBP), albumin (AB), N-acetyl-β-D-glucosaminidase (NAG), alkaline phosphatase (ALP), γ-glutamyl transpeptidase (GGT) and kidney injury molecule-1 (KIM-1). Spearman’s rank correlation was carried out to assess pairwise bivariate associations between continuous variables. Three different models of multiple linear regression (the *cre*-corrected, un-corrected and *cre*-adjusted model) were used to model the dose-response relationships between U-Cd and nine urine markers. *Results:* Spearman’s rank correlation showed that NAG, ALP, RBP, β_2_-MG and MT were significantly associated with U-Cd for both *cre*-corrected and observed data. Generally, NAG correlated best with U-Cd among the nine biomarkers studied, followed by ALP and MT. In the un-corrected model and *cre*-adjusted model, the regression coefficients and *R^2^* of nine biomarkers were larger than the corresponding values in the *cre*-corrected model, indicating that the use of observed data was better for investigating the relationship between biomarkers and U-Cd than *cre*-corrected data. *Conclusions:* Our results suggest that NAG, MT and ALP in urine were better biomarkers for long-term environmental cadmium exposure assessment among the nine biomarkers studied. Further, data without normalization with creatinine show better relationships between cadmium exposure and renal dysfunction.

## 1. Introduction

Cadmium (Cd) is one of the most toxic heavy metals originating from industrial leakage or from natural abundance; thus, the need for biomonitoring remains imperative in many countries (e.g., Sweden, [1,2] the USA [3], China [4,5], Japan [6,7]). Cd in the environment can be taken up via crops (such as grain and tobacco) and aquatic organisms (such as shellfish) and also through the food-chain [8]. For non-occupational groups, diet is a primary exposure source for non-smokers [9,10,11], and tobacco smoke is an important source of Cd for smokers; the intake from smoking may exceed that from food in the case of heavy smokers [8,12]. Huang, M. *et al.* found that the daily Cd intake from the diet was established as 7.07 μg/day in the general population from Korea [13]. Because of its long biological half-life (10–30 years), cadmium can be accumulated in some tissues or organs, age-dependently (particularly, kidney, liver and muscle) [8]. Cd in kidneys accounts for about 50% of body burden, and kidneys are the critical organs for cadmium toxic effects.

Numerous studies have shown that urinary excretion of cadmium (U­Cd) is a reliable indicator of excessive cadmium exposure and body burden [1,6,14]. In the general population from Korea, the mean concentration of Cd measured in urine was 0.95 μg/g creatinine and 0.84 μg/g creatinine [13,15], and the geometric mean for Cd was 1.6 μg/g creatinine in the general population from Japan [16]. Some studies indicate that smoking status and sex differences may be the important determinants of U­Cd [17]. Females may be an at-risk group compared to males because women tend to have higher U­Cd than men. This phenomenon can be explained by female low iron status, which is linked to increased intestinal absorption of Cd [17,18,19]. In addition, U­Cd was higher in smokers compared to non-smokers [17,20].

The primary toxic effect resulting from chronic Cd exposure is proximal renal tubular dysfunction, namely “cadmium nephropathy”, which is diagnosed by increased excretion of low molecular weight proteins in urine, such as β_2_-microglobulin (β_2_-MG) and retinol-binding protein (RBP), as well as α_1_-microglobulin (α_1_-MG) and metallothionein (MT), which are used as a measure of the effects on kidney function [1,8,21,22]. There are other biomarkers used as a measure of the effects on the kidney, like urinary enzymes N-acety1-β-glucosaminidase (NAG) and alkaline phosphatase (ALP), as well as high molecular weight proteins urinary albumin (AB) [4,14,16,23,24]. Recently, there were studies using other biomarkers for evaluating renal function, such as γ-glutamyltransferase (GGT) [25] and kidney injury molecule-1 (KIM-1) [26]. A number of studies have reported the relationship between U-Cd and the above-mentioned renal biomarkers, and among them, β_2_-MG was used most frequently. However, there have been inadequacies in some of these studies. For instance, only two or three conventional biomarkers are typically studied [1,3,4,5,14,16,17,27,28]. In addition, the difference between using creatinine-corrected and un-corrected data is crucial for the analysis of urine data, as creatinine-correction is often used to correct for urine dilution [29,30,31,32]. In particular, Barr *et al.* point out the salient and fundamental flaw in using creatinine-corrected data and suggest using creatinine as a predictor variable in linear models instead [29]. Nonetheless, all three methods of using creatinine data (*i.e.*, *cre*-corrected, un-corrected, *cre*-adjusted) have been used to evaluate the utility of biomarkers for Cd exposure, implying a lack of consensus in the literature.

Therefore, the aim of our study was to carry out a systematic investigation of the association between urinary excretion of Cd and nine renal biomarkers, consisting of the four more commonly studied biomarkers, namely NAG, α_1_-MG, β_2_-MG, MT, and five other biomarkers known to be associated with renal impairment, which were ALP, RBP, AB, GGT and KIM-1. In addition, we evaluated the difference between the three approaches of using creatinine data, by comparing the correlations and models with the best fit.

## 2. Materials and Methods

### 2.1. Study Populations and Biological Materials

All subjects gave their informed consent for inclusion before they participated in the study. The study was conducted in accordance with the Declaration of Helsinki, and the protocol was approved by the Ethics Committee of Guangdong Provincial Center for Disease Control and Prevention. During 2011 to 2012, after obtaining the participants’ informed consent by our investigators going door to door, we also obtained information from two rural areas with different cadmium contamination in southern China (one unpolluted area and one cadmium-polluted areas, in April to July 2012), where we recruited 225 non-smoking female farmers (age ranging from 44 to 71 years). Each subject provided her written informed consent, filled questionnaires (on life-style factors, including age, years of residence (YOR), height, weight, basic health conditions, *etc.*). Morning spot urine samples were collected for detecting urinary metal and urinary creatinine (U-*cre*), as well as nine urinary proteins and enzymes, including NAG, ALP, GGT, α_1_-MG, β_2_-MG, MT, KIM-1, RBP and AB. In addition, the body mass index (BMI) was computed from height and weight using the equation BMI = weight in kg/(height in m)^2^. We excluded subjects who had a history of diabetes or other chronic diseases that are likely to impact on renal function and those who had been occupationally exposed to Cd. Subjects that provided an insufficient volume of urine sample for lab assays and that had incomplete answers in questionnaires were also excluded. These exclusions resulted in final counts of 40, and 185 subjects were included in the final statistical analysis.

### 2.2. Laboratory Methods

More than 100 mL of spot urine samples were collected from each subject in polyethylene bottles and stored at 4 °C. The urine collection containers were pre-screened for Cd contamination before the investigation. We randomly selected 20 containers for Cd contamination. The containers were filled with 5% HNO_3_ solution (v:v) overnight, and Cd concentrations in the solution were determined. The Cd concentrations of all of the containers tested were below the limit of detection. The specimens were transported to Guangdong Provincial Center for Disease Control and Prevention (GDCDC) within 4 h, making sure that metals and biochemical indicators in urine were detected within 8 h. Urine of 0.5 mL exactly was drawn and mixed with 1% HNO_3_ (v:v), before the system was diluted to 5 mL. After the parameters were set with tuners, the quality control samples (Seronorm^TM^ Trace Elements Urine, Seronorm^TM^ Trace Elements Urine blank, Bio-Rad Level 2), blank samples and subjects’ samples were measured by means of inductively-coupled argon plasma mass spectrometry with an Agilent 7700 instrument (Agilent Technologies. Santa Clara, California, USA). A quality control sample was assayed after every 10 test samples, as described by Gao *et al.* [33]. The limit of detection (LOD) for U-Cd was 0.02 μg/L. The Cd concentrations for Seronorm^TM^ Trace Elements Urine and Seronorm^TM^ Trace Elements Urine blank were 4.4 ± 0.4 μg/L (the reference value was 4.6 ± 0.4 μg/L with an acceptable range of 3.8 to 5.4 μg/L) and below LOD, respectively. The immunoturbidimetric test was applied to determine the concentrations of β_2_-MG, α_1_-MG, AB and RBP in urine by using an automatic biochemistry analyzer (Hitachi 7600-010). The LOD for β_2_-MG, α_1_-MG, AB and RBP was 0.1 mg/L. The colorimetric assay in accordance with a standardized method was employed to detect the levels of ALP, GGT and NAG also by using an automatic biochemistry analyzer. The LOD for ALP, GGT and NAG was 1 U/L. MT and KIM-1 were measured by enzyme-linked immunosorbent assay (ELISA). The LOD for MT and KIM-1 was 0.1 μg/L and 1 ng/L. The urinary creatinine (U-*cre*) level was assayed by the picric acid method. The LOD for U-*cre* was 0.01 mmol/L.

### 2.3. Statistical Analysis

Analyses were undertaken using the statistical computing language R (v3.0.1) in an Integrated Development Environment (IDE) Rstudio (v0.97.551). All biological parameters were reported as median and interquartile range (25th percentile-75th percentile) and were log­transformed to an approximate normal distribution. Values below the limit of detection (LOD) were replaced with LOD/$\sqrt{2}$ in the calculation. Spearman’s rank correlation was carried out to assess pairwise bivariate associations between continuous variables. Three different multiple linear regression models, namely *cre*-corrected model, un-corrected model and *cre*-adjusted model, were performed to model the dose-response relationships between U-Cd (independent variable) and nine renal biomarkers (dependent variable). In the first model (*cre*-corrected model), U­Cd and biomarkers were expressed as per gram of U-*cre*, and the model was adjusted for age, YOR and BMI. In the second model (un-corrected model), U­Cd and biomarkers were expressed as per liter, namely observed values, and the model was adjusted for age, YOR and BMI. In the third model (*cre*-adjusted model), U­Cd and biomarkers were also expressed with observed values, but U-*cre* was treated to be a confounder, as well as age, YOR and BMI, which were adjusted in the model. The partial regression coefficient, 95% confidence interval and adjusted *R^2^* were reported and the regression models were assessed using graphical plots and residual diagnostics. A cut-off for statistical significance was set at *p* < 0.05. 

## 3. Results

A total of 185 subjects with complete information were included in the final statistical analysis. Most of the subjects were in their mid-50s (the median age of the subjects was 55.65 years), and the median years of residence (YOR) was 45.50 years. The median U-Cd concentration of the subjects was 2.25 μg/L, and for none of the 185 samples (0%) was the U-Cd below the LOD. The percentages below LOD for β_2_-MG, α_1_-MG, AB and RBP were: 27.6%, n = 51; 44.3%, n = 82; 13.5%, n = 25; 51.4%, n = 95, respectively. The percentages below LOD for ALP, GGT and NAG were: 5.95%, n = 11; 0.0%, n = 0; 0.0%, n = 0, respectively. The percentages below LOD for MT and KIM-1 were: 0.0%, n = 0; 0.0%, n = 0, respectively. The percentages below LOD for U-*cre* were 0.0%, n = 0. The concentrations of urinary creatinine and nine renal biomarkers were also shown (Table 1).

**Table 1:** Characteristics of the study participants (n = 185).

Variables	Median	P_25_ to P_75_
Age (years)	55.65	49.30 to 62.21
YOR (years)	45.50	31.00 to 56.00
BMI	21.50	19.90 to 24.78
U-*cre* (g/L)	0.82	0.51 to 1.19
U-Cd (μg/L) *	2.25	1.20 to 5.10
NAG (U/L) *	5.10	3.30 to 7.25
ALP (U/L) *	4.00	2.00 to 6.00
GGT (U/L) *	20.00	11.50 to 29.00
α_1_-MG (mg/L) *	0.31	0.07 to 6.49
β_2_-MG (mg/L) *	0.11	0.07 to 0.28
MT (μg/L) *	3.03	1.69 to 5.14
KIM-1 (ng/L) *	62.74	57.89 to 67.43
RBP (mg/L) *	0.07	0.07 to 0.14
AB (mg/L) *	2.86	0.46 to 7.03

AB, albumin; ALP, alkaline phosphatase; BMI, body mass index; GGT, γ-glutamyl transpeptidase; KIM-1, kidney injury molecule-1; MT, metallothionein; NAG, N-acetyl-β-D-glucosaminidase; RBP, retinol binding protein; U-Cd, urinary cadmium; U-cre, urinary creatinine; YOR, years of residence; α_1_-MG, α_1_-microglobulin; β_2_-MG, β_2_-microglobulin. * Metals and biomarkers in urine were observed values.

Spearman’s rank correlation coefficients (*r_s_*) between urinary metals and nine renal biomarkers were examined in a total of 185 cases. The results with *cre*-corrected values are shown in Table 2, while those with the observed values are shown in Table 3. For the observed data, with the exception of α_1_-MG and KIM-1, the other seven biomarkers were significantly correlated with U-Cd. Compared to the observed data, fewer biomarkers showed significant associations with U-Cd of the *cre*-corrected data, and the Spearman’s rank correlation coefficients also tend to be smaller than those of the observed data (Table 2). Among the four commonly-studied biomarkers, namely NAG, α_1_-MG, β_2_-MG and MT, only α_1_-MG appeared to show no significant associations with U-Cd for both *cre*-corrected and observed data, neither did KIM-1 (Table 2 and Table 3).

**Table 2:** Spearman’s rank correlation based on Cr-corrected data.

Variables	Cd	NAG	ALP	GGT	α_1_-MG	β_2_-MG	MT	KIM-1	RBP	AB
Age	−0.194 **^b^**	0.115	−0.187 **^§^**	−0.076	0.381 **^†^**	0.051	−0.176 **^§^**	0.183 **^§^**	−0.128	−0.03
YOR	0.074	0.132	0.106	−0.087	0.200 **^‡^**	0.172 **^§^**	0.173 **^§^**	0.091	0.166 **^§^**	0.148 **^§^**
BMI	−0.221 **^b^**	0.089	−0.191 **^‡^**	0.026	0.081	−0.05	−0.116	−0.007	−0.065	0.079
U-*cre*	−0.092	−0.412 **^†^**	−0.041	0.021	−0.214 **^‡^**	−0.219 **^‡^**	−0.399 **^†^**	−0.970 **^†^**	−0.437 **^†^**	0.189 **^§^**
Cd *	1	0.314 **^†^**	0.416 **^†^**	−0.113	−0.017	0.145 **^§^**	0.321 **^†^**	0.137	0.345 **^†^**	0.075
NAG *		1	0.396 **^†^**	0.11	0.356 **^†^**	0.365 **^†^**	0.279 **^†^**	0.429 **^†^**	0.345 **^†^**	0.284 **^†^**
ALP *			1	0.145 **^§^**	0.028	0.347 **^†^**	0.318 **^†^**	0.08	0.371 **^†^**	0.311 **^†^**
GGT *				1	−0.102	−0.085	−0.161 **^§^**	−0.041	−0.082	0.172 **^§^**
α_1_-MG *					1	0.280 **^†^**	0	0.187 **^§^**	0.051	0.357 **^†^**
β_2_-MG *						1	0.237 **^‡^**	0.243 **^†^**	0.314 **^†^**	0.312 **^†^**
MT *							1	0.426 **^†^**	0.516 **^†^**	0.105
KIM-1 *								1	0.474 **^†^**	−0.160 **^§^**
RBP *									1	0.008
AB *										1

AB, albumin; ALP, alkaline phosphatase; BMI, body mass index; GGT, γ-glutamyl transpeptidase; KIM-1, kidney injury molecule-1; MT, metallothionein; NAG, N-acetyl-β-D-glucosaminidase; RBP, retinol binding protein; U-Cd, urinary cadmium; U-cre, urinary creatinine; YOR, years of residence; α_1_-MG, α_1_-microglobulin; β_2_-MG, β_2_-microglobulin. * Metals and biomarkers in urine were corrected for creatinine concentration; **^†^** *p* < 0.001; **^‡^** *p* < 0.01; **^§^** *p* < 0.05.

**Table 3:** Spearman’s rank correlation based on observed data.

Variables	Cd	NAG	ALP	GGT	α_1_-MG	β_2_-MG	MT	KIM-1	RBP	AB
Age	−0.323 ^†^	−0.091	−0.310 ^†^	−0.235 ^‡^	0.313 ^†^	−0.019	−0.308 ^†^	−0.057	−0.251 ^†^	−0.079
YOR	−0.024	0.065	0.018	−0.086	0.178 ^§^	0.14	0.147 ^§^	0.074	0.143	0.092
BMI	−0.153 ^§^	0.03	−0.184 ^§^	−0.03	0.071	−0.07	−0.14	−0.139	−0.104	0.063
U-*cre*	0.719 ^†^	0.580 ^†^	0.564 ^†^	0.885 ^†^	0.127	0.078	0.125	−0.118	0.064	0.437 ^†^
Cd *	1	0.598 ^†^	0.641 ^†^	0.611 ^†^	0.077	0.161 ^§^	0.295 ^†^	0.057	0.320 ^†^	0.388 ^†^
NAG *		1	0.603 ^†^	0.564 ^†^	0.307 ^†^	0.283 ^†^	0.151 ^§^	0.082	0.217 ^‡^	0.570 ^†^
ALP *			1	0.593 ^†^	0.099	0.314 ^†^	0.290 ^†^	0.100	0.351 ^†^	0.493 ^†^
GGT *				1	0.074	0.069	0.054	−0.136	0.024	0.456 ^†^
α_1_-MG *					1	0.273 ^†^	−0.083	−0.075	−0.026	0.416 ^†^
β_2_-MG *						1	0.175 ^§^	0.103	0.269 ^†^	0.357 ^†^
MT^*^							1	0.180 ^§^	0.427 ^†^	0.207 ^‡^
KIM-1 *								1	0.219 ^‡^	0.057
RBP *									1	0.123
AB *										1

AB, albumin; ALP, alkaline phosphatase; BMI, body mass index; GGT, γ-glutamyl transpeptidase; KIM-1, kidney injury molecule-1; MT, metallothionein; NAG, N-acetyl-β-D-glucosaminidase; RBP, retinol binding protein; U-Cd, urinary cadmium; U-cre, urinary creatinine; YOR, years of residence; α_1_-MG, α_1_-microglobulin; β_2_-MG, β_2_-microglobulin. * Metals and biomarkers in urine were corrected for creatinine concentration; ^†^ *p* < 0.001; ^‡^ *p* < 0.01; **^§^** *p* < 0.05.

Multiple regression analysis was conducted with three different models (*cre*-corrected model, un-corrected model and *cre*-adjusted model) to describe the linear relationship between U-Cd and nine biomarkers. All three different models were adjusted for age, YOR and BMI, while the *cre*-adjusted model was also adjusted for creatinine concentration. The results demonstrated that all biomarkers showed significant associations with U-Cd in three different models, except for α_1_-MG, KIM-1 and AB. There was no significant association between α_1_-MG and U-Cd in any of the models. Moreover, KIM-1 only showed a relatively small association with U-Cd for *cre*-adjusted data (*β* = 0.177, *p* = 0.045), while AB was significant for the observed data only (*β* = 0.269, *p* = 0.001). In the *cre*-corrected model, NAG correlated most closely with U-Cd among the nine renal markers studied (*β* = 0.362, *p* < 0.001), followed by RBP (*β* = 0.362, *p* < 0.001) and ALP (*β* = 0.266, *p* < 0.001), but the *R^2^* were small in all biomarkers. 

Compared to the *cre*-corrected model, the un-corrected model clearly showed that the observed data were beneficial in obtaining larger partial regression coefficients. The biomarkers with regression coefficients exceeding 0.25 were NAG (*β* = 0.505, *p* < 0.001), ALP (*β* = 0.401, *p* < 0.001), RBP (*β* = 0.278, *p* < 0.001), AB (*β* = 0.269, *p* = 0.001) and MT (*β* = 0.261, *p* < 0.001). The *R^2^* of nine biomarkers were larger than those in the Cr-corrected model, as well. However, RBP had the largest percentage of subjects with levels below LOD (51.4%, n = 95), unlike ALP and AB with the percentage LOD below 15% (5.95%, n = 11; 13.5%, n = 25, respectively). 

Similar to the un-corrected model, the biomarkers with regression coefficients exceeding 0.25 in the *cre*-adjusted model were NAG (*β* = 0.344, *p* < 0.001), RBP (*β* = 0.312, *p* < 0.001), ALP (*β* = 0.272, *p* < 0.001) and MT (*β* = 0.263, *p* = 0.001). When the U-*cre* was also used as a predictor variable, it generally increased the *R^2^* in the model, with the exception of MT and KIM-1. The results showed that the regression coefficients and *R^2^* were higher in the un-corrected model and the *cre*-adjusted model compared to the *cre*-corrected model (Table 4).

## 4. Discussion

The purpose of the present study was to identify the suitable biomarkers among the above-mentioned nine that correlated most closely with Cd in the urine of residents with cadmium exposure. While blood Cd is considered to reflect current Cd exposure, U­Cd is widely used in biomonitoring and risk assessment studies as a classical biomarker of long-term exposure to Cd [8,34]. However, the U­Cd level may be affected by smoking status and gender differences, as reported in most earlier studies [17,18,19,20]. Further, exposure markers do not usually reflect the pathological changes of exposed persons. Therefore, in order to minimize the effect of known confounders, we recruited only non-smoking female farmers in our research. The motivation of discovering sensitive biomarkers related to Cd exposure for biomonitoring is due to kidney being the critical target organ of Cd exposure, and various histopathological changes occur after long-term Cd exposure, including epithelial cell damage in the proximal tubule, interstitial fibrosis and glomerular basal cell damage [8]. The earliest adverse effect of Cd is often manifested as an increased urinary excretion of low molecular weight proteins and enzymes (e.g., NAG, RBP, α_1_-MG, β_2_-MG, *etc.*) [8]. However, there is still a lack of consensus regarding which biomarker is most suitable to appropriately characterize renal function in populations chronically exposed to a low concentration of cadmium.

**Table 4:** Multiple linear regression analysis with nine renal markers as dependent variables based on three models.

Variables	Model	Coefficient	95% CI	*P*	*R^2^*
NAG	*cre*-corrected *	0.362	0.217,0.507	<0.001	0.179
	Un-corrected ^**†**^	0.505	0.361,0.649	<0.001	0.27
	*cre*-adjusted ^**‡**^	0.344	0.206,0.482	<0.001	0.413
ALP	*cre*-corrected *	0.266	0.117,0.415	<0.001	0.139
	Un-corrected ^**†**^	0.401	0.253,0.549	<0.001	0.234
	*cre*-adjusted ^**‡**^	0.272	0.124,0.420	<0.001	0.324
GGT	*cre*-corrected *	−0.186	−0.345,−0.028	0.023	0.021
	Un-corrected ^**†**^	0.236	0.084,0.388	0.003	0.194
	*cre*-adjusted ^**‡**^	−0.093	−0.173,−0.013	0.023	0.803
α_1_-MG	*cre*-corrected *	0.028	−0.121,0.178	0.713	0.127
	Un-corrected ^**†**^	0.116	−0.045,0.277	0.158	0.098
	*cre*-adjusted ^**‡**^	0.031	−0.136,0.198	0.718	0.134
β_2_-MG	*cre*-corrected *	0.168	0.010,0.326	0.038	0.029
	Un-corrected ^**†**^	0.221	0.056,0.384	0.01	0.053
	*cre*-adjusted ^**‡**^	0.176	0.002,0.352	0.049	0.058
MT	*cre*-corrected *	0.242	0.097,0.387	0.001	0.18
	Un-corrected ^**†**^	0.261	0.115,0.407	<0.001	0.251
	*cre*-adjusted ^**‡**^	0.263	0.107,0.419	0.001	0.247
KIM-1	*cre*-corrected *	0.084	−0.056, 0.224	0.243	0.235
	Un-corrected ^**†**^	0.098	−0.067, 0.262	0.247	0.053
	*cre*-adjusted ^**‡**^	0.177	0.005, 0.349	0.045	0.084
RBP	*cre*-corrected *	0.281	0.131,0.429	<0.001	0.137
	Un-corrected ^**†**^	0.278	0.121,0.435	<0.001	0.142
	*cre*-adjusted **^‡^**	0.312	0.145,0.479	<0.001	0.144
AB	*cre*-corrected *	0.092	−0.066,0.246	0.258	0.054
	Un-corrected **^†^**	0.269	0.109,0.429	0.001	0.101
	*cre*-adjusted **^‡^**	0.107	−0.049,0.263	0.181	0.244

AB, albumin; ALP, alkaline phosphatase; GGT, γ-glutamyl transpeptidase; KIM-1, kidney injury molecule-1; MT, metallothionein; NAG, N-acetyl-β-D-glucosaminidase; RBP, retinol binding protein; α_1_-MG, α_1_-microglobulin; β_2_-MG, β_2_-microglobulin. * The *cre*-corrected model stands for multiple linear regression adjusted for age, BMI and YOR, based on values corrected for creatinine concentration; ^†^ the un-corrected model stands for multiple linear regression adjusted for age, BMI and YOR, based on observed (un-corrected) values; ^‡^ the *cre*-adjusted model stands for multiple linear regression adjusted for age, BMI, YOR and creatinine concentration, based on observed (un-corrected) values.

In this study, we evaluated the relationship between environmental Cd exposure and various renal markers in non-smoking females aged 44 to 79 years in rural areas of China. Among the nine urinary renal markers investigated, we found that NAG, β_2_-MG, ALP, RBP and MT were able to show close associations with U-Cd for both *cre*-corrected and observed data in this in-depth study, on a homogenous population.

Additionally, NAG was shown to be the best biomarker among the widely available markers mentioned above with the largest regression coefficient and *R^2^* (*β* = 0.505, *p* < 0.001, *R^2^* = 0.270), followed by MT (*β* = 0.261, *p* < 0.001, *R^2^* = 0.251). These two biomarkers in our study were shown to be good biomarkers for Cd exposure, consistent with two previous studies [16,27]. NAG is a type of lysosomal enzyme existing in proximal tubular cells. When the proximal tubular epithelial cells are damaged or shed, NAG will be released into the tubular cavity and cause an increase in urinary excretion, but it is relatively stable in urine even without preservation [16,35]. Several studies suggested that β_2_-MG, which is the most widely-used biomarker, is not necessarily the best, and NAG could be superior to β_2_-MG for monitoring renal dysfunction for non-occupational populations after environmental Cd exposure [16,35,36,37]. In the present study, the greater regression coefficient of NAG than β_2_-MG indicated that the release of NAG from the damaged proximal tubular epithelial cell reflects Cd exposure more closely than the impaired function in reabsorbing β_2_-MG in this study population. Exposure to Cd induces MT in several tissues, including liver and kidneys, as shown in animals, as well as in humans. MT, as a low molecular weight heat-stable intracellular protein, served as an efficient intracellular scavenger, offering intracellular detoxification by binding to Cd [38]. MT is synthesized and binds with cadmium in liver upon cadmium induction. In tubular cells, cadmium MT is degraded and free cadmium ion released. Although tubular cells are able to synthesize MT themselves, the free cadmium will still exert adverse effects when it exceeds the synthetic capacity of tubular cells [38,39]. β_2_-MG, which is the most frequently-studied traditional biomarker of renal dysfunction [4,14,16,17,27,28,40,41], was suggested to be more informative than NAG as a prognostic indicator of renal function [4]. However, in our study, assays of RBP, α_1_-MG and β_2_-MG were apparently affected by the LOD of the measurements. The number of samples below LOD of RBP, α_1_-MG and β_2_-MG were 51.4% (n = 95), 44.3% (n = 82) and 27.6% (n = 51), respectively; hence, it was difficult to reflect their absolute values. This phenomenon has also been observed in previous studies. Pennemans *et al.* reported 84% and 28% missing values for α_1_-MG and β_2_-MG, respectively, while investigating their relationships with U-Cd during long-term, low-dose cadmium exposure, and they excluded both biomarkers from further analysis subsequently [26]. Similarly, Pless-Mulloli *et al.* reported 44.4% of samples with detected α_1_-MG showed concentrations near the limit of detection [42]. The high percentage of missing values of α_1_-MG and β_2_-MG thus affected their use as biomarkers for chronic Cd exposure, by the lack of stability of these biomarkers in acidic urine [43].

The use of ALP, GGT, KIM-1 and AB as renal markers for environmental Cd exposure had not been well investigated. In our study, we found that two of these biomarkers, namely ALP and RBP, were well associated with U-Cd after adjustments for confounders. In particular, ALP appeared to be a potential biomarker for Cd exposure, with high regression coefficients and *R^2^* in three models, respectively. Nonetheless, the ALP data were also affected by the LOD of the measurements (5.95%, n = 11). As a urinary enzymatic biomarker, ALP can be found in the brush border of the kidney. There are four isoenzymes of ALP in the system, which are not differentiated in the current study. However, because none of the subjects in our study were suffering from glomerulopathy, micro-albuminuria was nonexistent. Therefore, the observed urinary ALP was likely to originate from the kidney. There are also studies justifying that kidney is the origin of urinary ALP [21,22]. They elaborate that ALP is a leakage enzyme and appears in urine as a result of necrosis or apoptosis of the PCT cells. In PCT, the intestinal ALP (IALP) is an enzyme that can be found at the S3 segment, while the non-specific ALP (NSALP) can be found at the S1 and S2 segments. Additionally, urinary ALP has been reported as a biomarker of renal injury or renal diseases, such as acute kidney injury, chronic kidney disease and diabetic nephropathy [23,24,44]. Our research showed that ALP may be a potential biomarker that could be superior to traditional biomarkers (like α_1_-MG and β_2_-MG) for monitoring the health effects of general populations after environmental Cd exposure, but more investigations to elucidate the role it plays in Cd toxicokinetics are needed. Besides, KIM-1 only showed a small association with U-Cd for *cre*-adjusted data (*β* = 0.177, *p* = 0.045), while AB was significant for the observed data only (*β* = 0.269, *p* = 0.001). Moreover, GGT displayed a regression coefficient in different directions with three models. Our study suggested that they were not stable biomarkers associated with renal function.

Since many studies use U-*cre* to adjust the urine spot samples for dilution [4,17,27,41], concerns have also been expressed regarding the inadequacy of correction for U-*cre* concentration, because U-*cre* concentration could be affected by various factors, such as age, gender, diets, body composition, infections, *etc.* [28,29,32]. One of the problems associated with U-*cre* correction in our study is the age-dependent reduction in creatinine concentration, which was observed in some studies [37,45]. Moriguchi *et al.* reported that creatinine levels at the age of 60 years will be about two thirds of the levels at 30 years [37]. In the present study, women aged from 44 to 71 years were recruited, and using U-*cre* correction data was not appropriate in the population with such a wide range of ages. In our study, it was clear that using U-*cre* for correction does not improve the correlation of the effect markers with U-Cd, and the regression coefficients, as well as *R^2^* for the observed values were greater than counterpart values after correction for urine density in terms of U-*cre*. These results indicated that using creatinine to adjust for dilution may not be perfect, and the use of observed data was more informative for investigating the relationship between biomarkers and U-Cd. The same conclusion has been obtained from recent studies [16,26]. As a result, Barr *et al.*, 2005, suggest using creatinine as a predictor variable in linear models instead [29]. This is equivalent to statistically adjusting for urine dilution, instead of using U-*cre* as a correction factor.

Our results showed that using U-*cre* as a predictor performed in the model can increase the *R^2^* in multiple linear regression models. Most biomarkers showed the highest *R^2^* in the *cre*-adjusted model, followed by the un-corrected model, and the lowest for the *cre*-corrected model, implying that using creatinine as a predictor variable may be a good method of describing the relationship between U-Cd and biomarkers. As we chose non-smoking female farmers as research subjects, confounders like gender and smoking status have been well controlled. Moreover, the subjects of this study also were likely to reflect the rural population’s Cd exposure in China better.

## 5. Conclusions

In conclusion, the present in-depth study showed that, for monitoring of general populations for the effects of Cd exposure, NAG and MT were good biomarkers. In contrast, the use of α_1_-MG, β_2_-MG and RBP as biomarkers was limited by the high percentage of missing values, due to the LOD of the measurements. Nevertheless, ALP appears to be a potential biomarker for Cd exposure, but this still requires further investigations to elucidate the role that it plays in Cd toxic kinetics. We have also demonstrated that using U-*cre* to adjust for dilution may not be suitable, and the use of the observed data was more informative for investigating the relationship between biomarkers and U-Cd. In addition, it is advisable to include U-*cre* as a predictor variable in the linear model in order to better reflect the relationship between U-Cd and biomarkers. 
